# Cytomegalovirus infection and disease reduce 10-year cardiac allograft vasculopathy-free survival in heart transplant recipients

**DOI:** 10.1186/s12879-015-1321-1

**Published:** 2015-12-24

**Authors:** Inger Johansson, Rune Andersson, Vanda Friman, Nedim Selimovic, Lars Hanzen, Salmir Nasic, Ulla Nyström, Vilborg Sigurdardottir

**Affiliations:** Department of Infectious Diseases, Institute of Biomedicine, Sahlgrenska Academy, Gothenburg University, Gothenburg, Sweden; Transplant Institute, Sahlgrenska University Hospital, Gothenburg, Sweden; Research and Development Centre, Skaraborg Hospital, Skövde, Sweden; Department of Cardiology, Swiss Cardiovascular Centre, University Hospital (Inselspital Bern) and University of Bern, Bern, Switzerland

**Keywords:** Heart transplantation, CMV, Cardiac allograft vasculopathy, Survival

## Abstract

**Background:**

Cytomegalovirus (CMV) is associated with an increased risk of cardiac allograft vasculopathy (CAV), the major limiting factor for long-term survival after heart transplantation (HTx). The purpose of this study was to evaluate the impact of CMV infection during long-term follow-up after HTx.

**Methods:**

A retrospective, single-centre study analyzed 226 HTx recipients (mean age 45 ± 13 years, 78 % men) who underwent transplantation between January 1988 and December 2000. The incidence and risk factors for CMV infection during the first year after transplantation were studied. Risk factors for CAV were included in an analyses of CAV-free survival within 10 years post-transplant. The effect of CMV infection on the grade of CAV was analyzed.

**Results:**

Survival to 10 years post-transplant was higher in patients with no CMV infection (69 %) compared with patients with CMV disease (55 %; *p* = 0.018) or asymptomatic CMV infection (54 %; *p* = 0.053). CAV-free survival time was higher in patients with no CMV infection (6.7 years; 95 % CI, 6.0–7.4) compared with CMV disease (4.2 years; CI, 3.2–5.2; *p* < 0.001) or asymptomatic CMV infection (5.4 years; CI, 4.3–6.4; *p* = 0.013). In univariate analysis, recipient age, donor age, coronary artery disease (CAD), asymptomatic CMV infection and CMV disease were significantly associated with CAV-free survival. In multivariate regression analysis, CMV disease, asymptomatic CMV infection, CAD and donor age remained independent predictors of CAV-free survival at 10 years post-transplant.

**Conclusions:**

CAV-free survival was significantly reduced in patients with CMV disease and asymptomatic CMV infection compared to patients without CMV infection. These findings highlight the importance of close monitoring of CMV viral load and appropriate therapeutic strategies for preventing asymptomatic CMV infection.

## Background

Cardiac allograft vasculopathy (CAV) is a major limiting factor for long-term survival following heart transplantation (HTx) [1]. CAV is a complex, multifactorial process with various immunological and non-immunological risk factors, including older donor age, pre-transplant coronary artery disease (CAD) and cardiovascular risk factors, implicated in its pathogenesis [1–4].

Cytomegalovirus (CMV) infection may play an essential role in CAV progression [5–7]. Following primary infection, CMV remains latent in CD 34+ bone marrow progenitor cells and monocytes [8] Cytomegalovirus reactivates frequently. The endothelial cell appears to be a target for CMV. Evidence of a link between CMV and CAV has been described [9–11], but other studies have not confirmed these findings [12, 13]. The development of CAV is initiated within the first year after transplantation [14] and the highest incidence of CMV infection occurs during the same period. In a recent study, asymptomatic CMV infection was shown to affect up to 50 % of patients, but the incidence of CMV disease was low with pre-emptive therapy [15]. However, the impact of asymptomatic CMV infection on the long-term progression of CAV has not been studied.

The primary aim of our study was to investigate the impact of asymptomatic CMV infection and CMV disease on overall survival and CAV during long-term follow up after heart transplantation. In addition, the study evaluated potential predictors for CAV-free survival and severity of CAV.

## Methods

### Study population

This was a retrospective, single-centre study of all patients undergoing first heart transplantation in Gothenburg, Sweden, between January 1988 and December 2000. During this period, 283 transplantations were performed in 278 patients. Of these, 34 patients died within 30 days of transplantation and were excluded from the analysis, as were 17 children < 14 years and one patient for whom the record from 1988 could not be found. In 226 patients, CMV infection, disease and potential risk factors were evaluated during the first 12 months after transplantation. Angiographic signs of CAV were recorded for 10 years or until death. Cardiovascular risk-factors were monitored according to protocol. Statins was given as universal therapy after 1996. Follow-up of all patients was complete through 31 December 2000. No patients were lost to follow-up.

### Immunosuppressive therapy

The induction therapy during 1988–1993 consisted of cyclosporine A (CsA) and from 1994 of anti-thymocyte globulin (ATG; 2.5 mg/kg/day administered intravenously before surgery and for 3 to 5 days afterwards). Daclizumab or 100 mg of prednisone was given in the event of ATG allergy. Methylprednisolone was administered at 500 mg intravenously before surgery and 500 mg intra-operative and then at 125 mg every 8 hours for 3 doses during the whole study period. The maintenance immunosuppression therapy consisted of standard triple therapy. CsA (5 to 8 mg/kg/day) was used to maintain serum CsA levels within range 200–350 ng/ml during the first year and from 100 to 200 ng/ml thereafter. Azathioprine (AZA) was administered at 2 mg/kg/day, and prednisone at 0.2 mg/kg/day reduced to 0.1 mg/kg/day orally. Since 1995, Tacrolimus (TAC) was an alternative to CsA, given at 0.075 mg/kg to maintain serum tacrolimus levels within range 10–15 ng/ml and, since 1997, AZA was replaced by mycophenolate mofetil (MMF) (2–3 g/day).

### Detection of CMV infection

CMV infection was detected by serology (seroconversion post-transplant), viral culture, qualitative polymerase chain reaction (PCR) for CMV DNA, histopathology and immunohistochemistry (IHC) with CMV-specific antibodies from endomyocardial biopsies (EMB) or tissue biopsies from other organs. Clinical symptoms of CMV disease were also documented for 12 months after transplantation.

### Definitions of CMV infection

*CMV infection*: CMV virus detected by viral culture or qualitative, PCR assay for CMV in any body fluid or tissue specimen. Seroconversion from CMV (seronegative to seropositive) was also regarded as CMV infection.

CMV infection was categorised as either asymptomatic CMV infection or CMV disease.

*Asymptomatic CMV infection*: Evidence of CMV infection but not fulfilling criteria for CMV disease.

*CMV disease*: Evidence of CMV infection with attributable symptoms in accordance with Ljungman et al. [16]. CMV disease was categorized as tissue-invasive disease or CMV syndrome with fever, leucopoenia and/or thrombocytopenia.

### Prophylaxis, treatment and monitoring of CMV

*All patients during 1988 to 1991*: No CMV prophylaxis was given. Patients were tested frequently. Seronegative patients received treatment with intravenous (i.v.) ganciclovir or foscavir for 14–21 days in the event of seroconversion or a positive viral culture for CMV.

*High-risk group (D+/R−) during 1992 to 1997*: Pre-emptive treatment was given, comprising monitoring with qualitative CMV PCR once weekly during the first three months post-transplantation. When CMV DNA was detected in serum, patients received treatment with i.v. ganciclovir for at least 10 days. *During 1998–2000*, universal prophylaxis was given with 1,000 mg of oral ganciclovir tid for 14 weeks.

*Intermediate-risk group (R+) during 1992 to 2000*: No prophylaxis was given. Qualitative CMV PCR was analyzed in serum when CMV disease was clinically suspected.

In D+/R−transplants, serological analyses were repeated once monthly for the first 4 months after HTx, then at 6, 9 and 12 months and thereafter annually and when infection was suspected during 1988 to 1998.

CMV disease was treated with 5 mg/kg of i.v. ganciclovir bid for 10–21 days. Asymptomatic CMV infection was treated in seronegative recipients. Ganciclovir dosing was adjusted for renal function. Patients who developed severe CMV pneumonitis (hypoxia) also received polyclonal immunoglobulin.

### Diagnosis of CAV

All available coronary angiographic studies performed in the study cohort between the first and tenth year of follow up, or until death or re-transplantation, were retrospectively re-analyzed visually. Where coronary angiography was not performed in patients who were alive during the study period it was due to medical contraindications or patient refusal. CAV-free survival was defined as the time to CAV of any grade observed visually by angiography. CAV was graded according to Costanzo et al. (none, mild, moderate or severe) [17]. The severity of CAV (none, mild, moderate, severe) was assessed throughout follow up. Donor-related coronary artery disease was defined as no stenosis = 0 or no significant stenosis = 1 from coronary angiographic studies performed before HTx.

### Risk factor analysis

Data on potential risk factors for CAV were collected retrospectively, including recipient and donor characteristics, cold ischemic time, cardiovascular risk factors (hypertension, diabetes mellitus, smoking status before transplantation and complications (acute rejection (AR) episodes).

Surveillance endomyocardial biopsies for AR were standardized for all patients and graded according to the 2005 ISHLT classification as 1R, 2R or 3R [18]. AR therapy consisted of 1,000 mg boluses of methylprednisolone for 3 consecutive days in cases of AR ≥ 2R. Severe cellular rejections were treated with 2.5 mg/kg/day of ATG for 3 days. Clinical relevant antibody-mediated rejection (AMR) was treated with plasmapheresis. The cumulative effect of acute cellular rejection was assessed by the total rejection score (TRS) [19] and defined as 0R = 0, 1R = 1, 2R = 2, 3R = 3. Severe TRS was defined as all AR ≥ 2R. The scores were normalized for the total number of biopsy specimens taken during the first 12 months after HTx (TRS or TRS ≥ 2R) in the individual patient.

### Ethics

This study was approved by the local Ethical Committee of Gothenburg (115–14) and by the medical director of the heart transplant department at the University Hospital, Gothenburg, Sweden. The data were recruited from the patients’ medical records and local registries according to standards by the Declaration of Helsinki.

### Statistical analyses

Data were analyzed using SPSS version 20.0 (SPSS Inc, Chicago, IL, US). Continuous variables are presented as mean values ± standard deviations (SD) and categorical variables as percentages. The chi-square test was used to compare proportions and occurrences between groups. Confidence intervals (CI) were calculated using a normality approximation algorithm. Survival and CAV-free survival time was analyzed using the Kaplan–Meier procedure and statistical comparisons of survival distributions between different categories were made using the log rank test. Cox’s univariate and multivariate model was used to determine risk factors for events. Variables in the univariate model testing with a p value < 0.1 were included in the multivariate model. A *p* value < 0.05 was considered statistically significant.

## Results

### Patient characteristics

In total, 226 patients who received a first HTx and had complete data regarding CMV infection during the first post-transplant year were included in the analysis. The baseline characteristics of recipients and donors are described in Table 1. The main causes of death were CAV (14 %), acute rejection (3 %) and infection (9 %). The mean follow-up for CAV disease was 8.9 years.

**Table 1:** Patient characteristics (*n* = 226)

Age at HTx, mean ± SD, years	
Recipient (R)	45 ± 13
Donor (D)	33 ± 12
Male gender, n (%)	
Recipient	176 (78)
Donor	159 (70)
Body mass index at HTx, mean ± SD kg/m^2^	23.8 ± 3.9
Cold ischemic time, mean ± SD minutes CMV serology, n (%)	168 ± 49
D+ /R−	46 (20)
D+ /R+	101 (45)
D− /R+	64 (28)
D− /R−	15 (7)
Induction therapy, n (%)	
CsA	48 (21)
ATG	165 (73)
Daclizumab	4 (2)
Prednisone	9 (4)
CAD, n (%)	73 (32)
Hypertension, n (%)	30 (13)
Diabetes Mellitus, n (%)	23 (10)
Previous smoking, n (%)	127 (56)
TRS at 1 year, mean ± SD	0.49 ± 0.30
TRS ≥ 2R at 1 year, mean ± SD	0.22 ± 0.20

Diagnosis of CMV infection or disease were performed by seroconversion in 25 patients, qualitative CMV PCR in 49 patients, tissue biopsies with histopathology and IHC with CMV-specific antibodies in 19 patients, and viral culture in 16 patients. CMV retinitis was diagnosed in one patient by an ophthalmologist.

### Incidence of CMV infection and disease

Of the 226 patients analyzed, 28 % (*n* = 64) developed CMV disease (tissue invasive, 11 % (*n* = 26) and 17 % (*n* = 38) developed CMV syndrome). Asymptomatic CMV infection was detected in 20 % of patients (*n* = 46).

In the 26 patients with tissue-invasive CMV disease, the following manifestations were recorded; myocarditis (*n* = 10), myocarditis + pneumonitis (*n* = 1), myocarditis + gastrointestinal disease (*n* = 1), gastrointestinal disease (*n* = 7), pneumonitis (*n* = 5), nephritis (*n* = 1) and retinitis (*n* = 1). The 26 cases of tissue-invasive CMV disease were proven in 85 % of the patients (*n* = 22) and possible in 15 % (*n* = 4).

The incidences of CMV disease and asymptomatic CMV infection according to the CMV serology status (donor positive (D+) or negative (D−) vs recipient positive (R+) or negative (R−)) are shown in Table 2. The onset of CMV disease in the high risk (D+/R−) serology group without CMV prophylaxis occurred significantly earlier (57 (22–178) days) after transplantation compared to the patients given oral ganciclovir for 14 weeks (103 (64–156) days) after transplantation (*p* = 0.008). There was no significant difference in the onset of CMV disease in the intermediate-risk serology groups (R+) without prophylaxis (45 (19–86) days) compared with targeted prophylaxis (51 (17–151) days) after transplantation (*p* = ns).

**Table 2:** Morbidity in CMV disease and asymptomatic CMV infection related to CMV serostatus

Serostatus	Number of patients	CMV disease, n (%)	Asymptomatic CMV infection, n (%)	No CMV, n (%)
D+/R−	46	30 (65)	11 (24)	5 (11)
D+/R+	101	21 (21)	19 (19)	61 (60)
D−/R+	64	11 (17)	12 (19)	41 (64)
D−/R−	15	2 (13)	4 (27)	9 (60)
Total	226	64 (28)	46 (20)	116 (51)

*D* donor; *R* recipient

The combined incidence of CMV disease or asymptomatic CMV infection was highest in the D+/R− group compared with the D+/R+, D−/R+ and D−/R− groups (*p* < 0.001) (Table 2). When the different eras of prophylaxis strategy were taken into account, there was no statistical significant difference in the incidence of CMV disease or asymptomatic infection compared to no CMV infection in the high risk (D+/R−) group (*p* = 0.08) (Table 3) or the intermediate-risk (R+) group (*p* = 0.25) (Table 4). In addition, across the total study group there was no statistical difference between different eras in terms of the incidence of CMV disease or asymptomatic CMV infection (*p* = 0.62) at 1 year after transplantation.

**Table 3:** Incidence of CMV disease and asymptomatic CMV infection in D+/R− transplants according to era after HTx

Era	Prophylaxis	CMV disease, n (%)	Asymptomatic CMV infection, n (%)	No CMV, n (%)
1988–1991 (*n* = 18)	No	12 (67)	5 (28)	1 (5)
1992–1997 (*n* = 17)	Pre-emptive^a^	13 (76)	3 (18)	1 (6)
1998–2000 (*n* = 11)	Universal^b^	5 (46)	3 (27)	3 (27)
Total (*n* = 46)		30 (65)	11 (24)	5 (11)

^a^Pre-emptive treatment, monitoring with qualitative CMV PCR once weekly or two weeks apart for the first three months post-transplantation ^b^1,000 mg of oral ganciclovir tid for 14 weeks

**Table 4:** Incidence of CMV disease and asymptomatic CMV infection in R+ recipients according to era after HTx

Era	Prophylaxis	CMV disease, n (%)	Asymptomatic CMV infection, n (%)	No CMV, n (%)
1988–1991 (*n* = 59)	No	11 (19)	8 (13)	40 (68)
1992–1997 (*n* = 74)	Targeted^a^	11 (15)	17 (23)	46 (62)
1998–2000 (*n* = 32)	Targeted^a^	10 (31)	6 (19)	16 (50)
Total (*n* = 165)		32 (19)	31 (19)	102 (62)

^a^Targeted prophylaxis given with 5 mg/kg of i.v. ganciclovir bid for 10 days in association with the first anti-rejection treatment with ATG and the second anti-rejection treatment with high-dose corticosteroids within the first 4 months post-transplantation

### Survival according to CMV status

There were no significant differences in the risk for CMV disease or asymptomatic CMV infection versus no CMV infection according to recipient age (*p* = 0.537), donor age (*p* = 0.072), TRS at 1 year post-transplant (*p* = 0.483) or TRS ≥ 2R score at 1 year (*p* = 0.259).

**Fig. 1:**
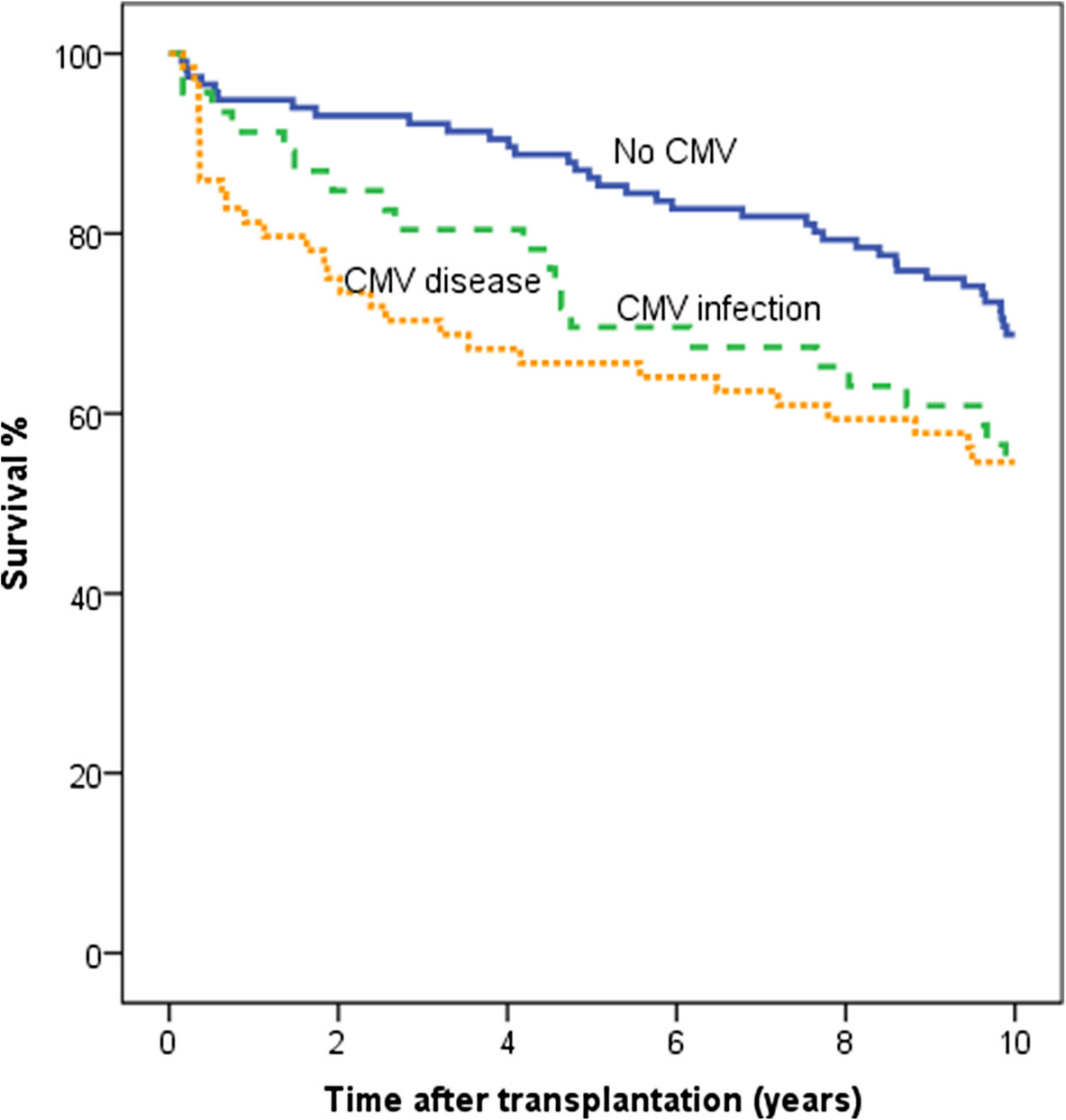
Survival—10 years of follow-up in 226 heart transplant recipients. Survival during a follow-up of 10 years was significantly higher for patients without CMV infection, *n* = 116 (69%), compared with patients with CMV disease, *n* = 64 (55%; *p* = 0.018), and asymptomatic CMV infection, *n* = 46 (54%; *p* = 0.053). (Patients were followed to re-transplantation (*n* = 5) or death) Kaplan–Meier survival curve, the mean follow-up was 9.9 years

Survival was 7.0 years (95 % CI 6.0–7.9) for patients with CMV disease, 7.5 years (95 % CI 6.4–8.5) with asymptomatic CMV infection and 8.7 years (95 % CI 8.2–9.2) with no CMV infection. Survival at 10 years post-transplant (mean 9.9 years) was significantly higher for patients without CMV infection (69 %), compared with patients who had CMV disease (55 %; *p* = 0.018) or asymptomatic CMV infection (54 %; *p* = 0.053) (Fig. 1).

### CAV-free survival and risk factors of CAV by univariate and multivariate analyses

A total of 1272 coronary angiographic studies performed in 204 patients between 1 and 10 years after HTx were re-analyzed. Twenty-two patients did not survive to the first coronary angiography. In the last coronary angiography performed, no CAV was found in 113 (50 %) patients, mild CAV in 40 (18 %) patients, moderate CAV in 41 (18 %) patients and severe CAV in 10 (4 %) patients. Sixty-four coronary angiographies were performed in donors before HTx. Thirty-one patients had CAV at the first year after HTx, of whom 6 had donor-related coronary artery disease and 8 had no lesions on angiography before HTx. The TRS at one year was 0.49 ± 0.30 and TRS ≥ 2R was 0.22 ± 0.20. Histologically suspected AMR was found in 26 patients, 5 of whom received plasmapheresis and 1 underwent re-transplantation due to graft loss.

**Fig. 2:**
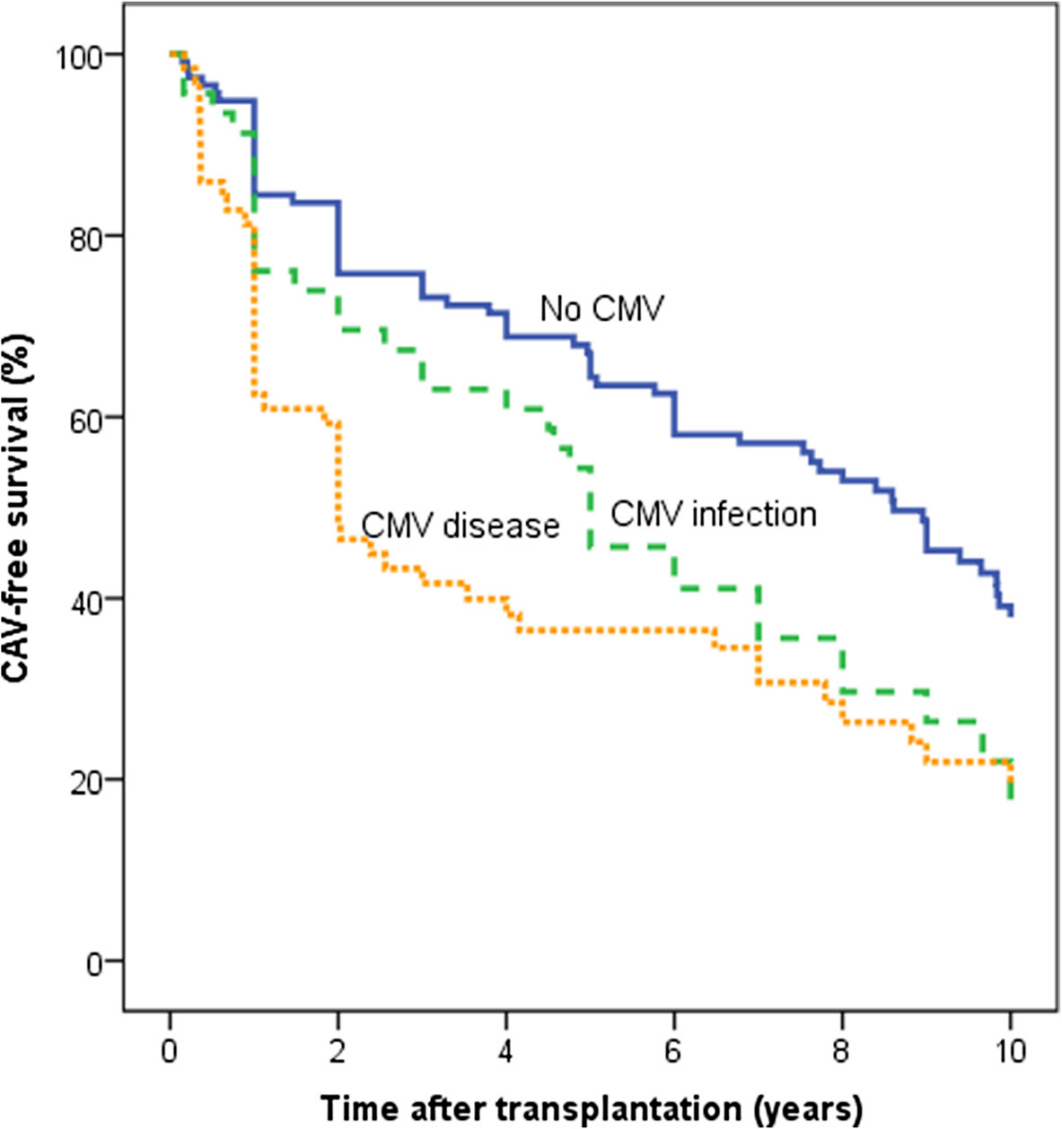
CAV-free survival—10 years of follow-up in 226 heart transplant recipients. CAV-free survival during a follow-up of 10 years was significantly higher for patients with no CMV infection (*n* =116) compared with patients with CMV disease (*n* = 64; *p* <0.001) and asymptomatic CMV infection (*n* = 46; *p* = 0.013). (Patients were followed to re-transplantation (*n* = 5) or death) Kaplan–Meier curve, the mean follow-up was 8.9 years

CAV-free survival for the total study population was 5.7 years (95 % CI 5.21–6.24). CAV-free survival was significantly longer for patients without CMV infection 6.7 years (95 % CI 6.0–7.4) compared with patients with CMV disease 4.2 years (95 % CI 3.2–5.2) (*p* < 0.001) or asymptomatic CMV infection 5.4 years (95 % CI 4.3–6.4), (p = 0.013) (Fig. 2).

Univariate analysis for potential risk factors for CAV or death (time to first event) during 10 years follow-up showed that recipient age, donor age, CAD, asymptomatic CMV infection, CMV disease and TRS ≥ 2R were statistically associated with CAV-free survival at 10 years (Table 5).

**Table 5:** Univariate analysis for risk factors associated with CAV-free survival 10 years after Htx

Risk factor	HR	95 % CI	*p*-value
Recipient male	1.18	0.79–1.76	0.417
Recipient age	1.01	1.00–1.03	0.034
Recipient body mass index	1.03	0.99–1.08	0.137
Donor male	1.13	0.79–1.63	0.491
Donor age	1.04	1.03–1.06	<0.001
CAD	1.65	1.18–2.30	0.003
No CMV	-	-	Ref.
CMV disease	2.03	1.39–2.95	<0.001
Asymptomatic CMV infection	1.63	1.07–2.46	0.022
Donor-related CAD	1.49	0.86–2.59	0.153
Cold ischemic time	1.00	0.99–1.00	0.518
AMR at first year	1.18	0.71–1.96	0.531
TRS	1.51	0.84–2.72	0.165
TRS ≥ 2R	2.07	0.87–4.93	0.099
Hypertension	1.39	0.89–2.18	0.146
Diabetes mellitus	1.05	0.85–1.30	0.646
Ex-smoker	1.23	0.87–1.76	0.245
CMV serology			
D−/R−	-	-	-
D−/R+	1.22	0.57–2.62	0.602
D+/R−	1.80	0.82–3.91	0.141
D+/R+	1.50	0.72–3.12	0.278

*Ref* reference category when calculating HR

In a multivariate Cox-regression analysis, CMV disease, asymptomatic CMV infection, CAD and donor age were independent predictors for CAV-free survival at 10 years after transplantation (Table 6).

**Table 6:** Multivariate analysis for risk factors associated with CAV-free survival 10 years after Htx

Risk factor	HR	95 % CI	*p*-value
Recipient age	1.00	0.98–1.01	0.424
Donor age	1.04	1.02–1.06	<0.001
CAD	1.86	1.19–2.93	0.007
TRS ≥ 2R	2.01	0.84–4.80	0.114
No CMV	-	-	Ref.
CMV disease	1.88	1.21–2.91	0.005
CMV infection	1.75	1.11–2.77	0.017

*Ref* reference category when calculating HR

**Fig. 3:**
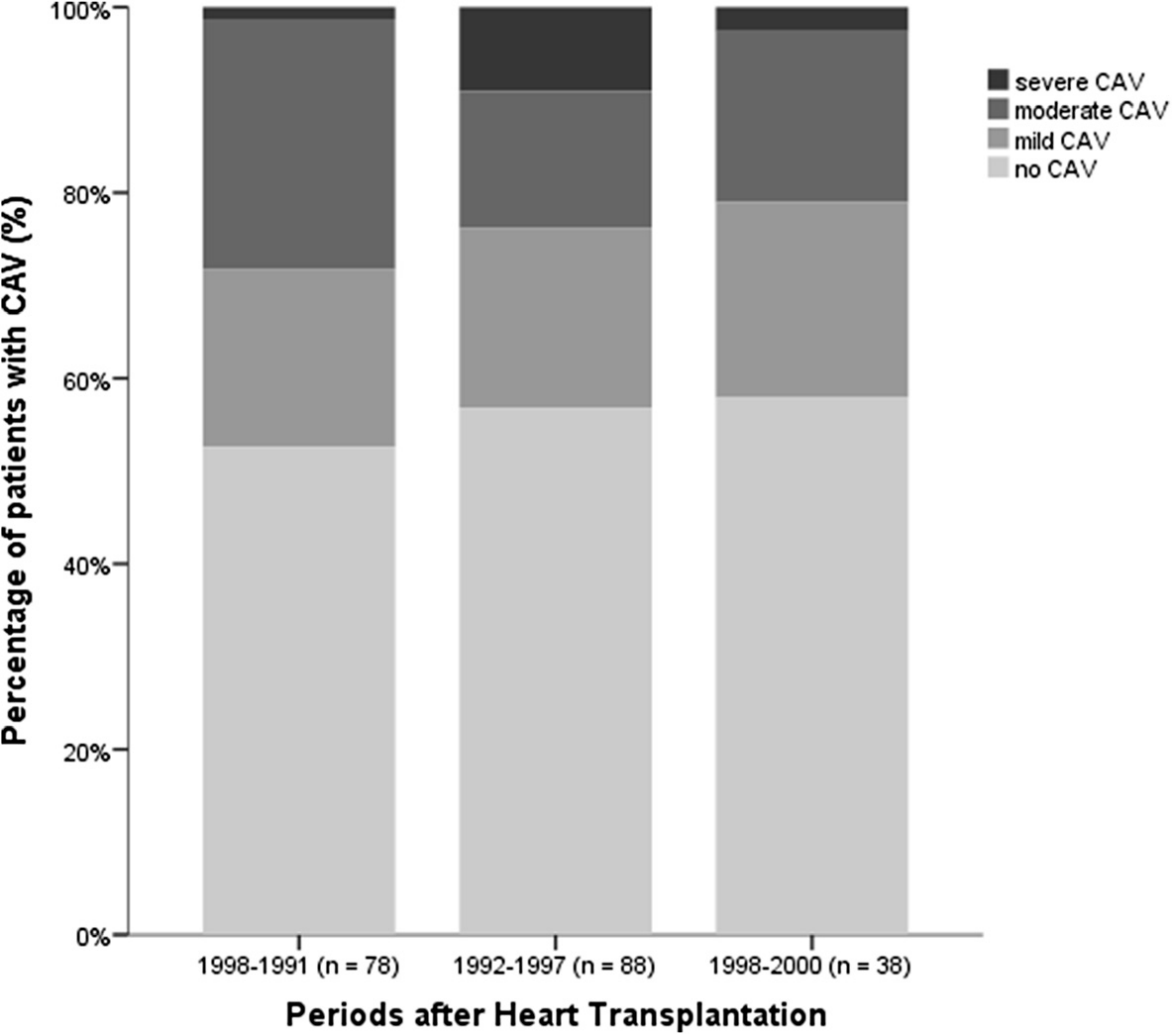
The grade of CAV according to different era after transplantation. Result of coronary angiographies showing the distribution in the grade of CAV in the different observation periods after heart transplantation (*p* = 0.175). CAV, cardiac allograft vasculopathy

There was no statistical significant difference in the grade of CAV at 10 years according to different eras of transplantation (*p* = 0.175) (Fig. 3) or CAV status (*p* = 0.81).

## Discussion

The main finding of this study is that not only CMV disease but also asymptomatic CMV infection during the first year after heart transplantation predispose patients to develop cardiac allograft vasculopathy over the long term. In addition, CMV disease was an independent predictor of survival after 10 years of follow-up.

In previous reports, CMV infection has repeatedly been shown to play an essential role in CAV progression [9, 10, 20, 21] although several studies have not found an association between CMV and CAV [12, 13, 22]. The reasons for this discrepancy could include inadequate sample size, short-term follow up, different diagnostic methods for CAV and varying definition for CMV infection. Sagerdal et al. showed that during long-term follow-up of kidney transplants, CMV disease or asymptomatic CMV infection within the first 100 days after kidney transplantation were independent risk factors for major cardiovascular events and mortality [23].

Our observations regarding the effect of CMV disease and asymptomatic CMV infection on long-term CAV-free survival after heart transplantation are in line with a recent study by Delgado et al. [24]. In their study, CMV infection was monitored during the first year after transplantation and both CMV disease and asymptomatic CMV viremia were shown to be independent predictors for long-term development of CAV. Both our analysis and that of Delgado et al. [24] included patients from the 1990s, when immunosuppressive treatment was intensive and monitoring strategies with limited CMV prophylaxis therapy were practised. These results support the emerging evidence that more aggressive monitoring and treatment strategies are important to prevent CMV infection. Potena et al. have shown that in heart transplant patients managed by a pre-emptive strategy, asymptomatic CMV infection was associated with an increased risk of developing CAV, defined as abnormal coronary remodeling 1 year after HTx [6]. In another report from Potena et al. CAV was reduced by the suppression of subclinical CMV infection [25], indicating not only an association but also a possible causal role for CMV in the pathogenesis of CAV. The finding that subclinical (i.e. asymptomatic) CMV infection is associated with CAV development is also consistent with the data showing that universal CMV prophylaxis is associated with less intimal thickening [26].

The link between AR and CAV is controversial [19, 24, 27–30]. Raichlin et al. showed that AR during the first 3 to 6 months after transplantation predisposed patients to onset of CAV [19]. Caforio et al. found that rejection score was an independent predictor of CAV onset, but not severity [30]. Delgado et al. also found that severe acute cellular rejection and donor age were independent predictors of CAV, consistent with the outcome of our univariate analyses [24]. However, only donor age and previous CAD remained as predictors of CAV in our multivariate analysis.

The strength of our study is the large monocentric study population (*n* = 226) and the long-term follow-up, with CAV-free survival follow-up over a mean of 8.9 years. A high proportion of the recipients (49 %; 110/226) were diagnosed with CMV disease or asymptomatic CMV infection, making it easier to study the long-term influence of CMV. The same person reviewed all the medical records. A diagnosis of CMV, and CMV-related symptoms, were carefully reviewed and re-evaluated throughout the follow up period making the diagnosis of asymptomatic CMV infection reliable.

Limitations are that this is a retrospective analysis of a heterogeneous patient cohort in which different diagnostic methods and prophylaxis strategies were applied during the observation period. However, after adjustments for the different periods after transplantation (i.e. based on prophylaxis strategy), we found no statistical significant differences in the incidence of CMV disease, asymptomatic CMV infection, CAV status or grade of CAV. In addition, a pathological diagnosis detected by IHC with CMV-specific antibodies was available at all times and was used more frequently than today. The qualitative CMV PCR and, later, quantitative PCR used in our study is more sensitive than viral culture and seroconversion, and makes it possible to treat CMV infection early, before CMV disease occurs. We might, however, have missed some patients with asymptomatic CMV infection since scheduled CMV screening was only performed in the high-risk serology group during the early part of the observation period. We did not include HLA mismatches or lipid levels as variables because of insufficient data. In a systematic review of factors associated with CAV, lipid levels were not shown to be associated with CAV using angiography [31]. Although the coronary angiography used in our study is not as sensitive as intravascular ultrasound, we were able to compare our results to the most recent evidence relating to the long-term effects of CMV infection on CAV using angiography [24]. In addition, coronary angiographies were frequently performed in our population and a cardiologist re-evaluated and graded all coronary angiographies according to the consensus paper of Costanzo et al. [17]. Donor-related CAD was assessed by coronary angiography before transplantation in only a subset of the study cohort. However, the mean age of the donors was low (33 years). We have previously shown that selection of donor hearts older than 40 years of age based on coronary angiography to exclude pre-existing CAD did not reduce the prevalence of CAD nor improved survival among heart recipients between 1988 and 2005 [32].

## Conclusions

In this long-term follow up of heart transplant recipients, CMV disease and asymptomatic CMV infection, together with donor age and previous CAD, were independent predictors of angiographic CAV. Our study supports emerging evidence that aggressive strategies to prevent not only CMV disease but also asymptomatic CMV infection may be important in reducing the early and late development of CAV. More studies are required to define the optimal length of CMV prophylaxis and the approach, i.e. pre-emptive vs universal prophylaxis, in order to prevent low-viral replication of CMV.

## Abbreviations

AMR: antibody-mediated rejection
AR: acute rejection
ATG: anti-thymocyte globulin
CAD: coronary artery disease
CAV: cardiac allograft vasculopathy
CMV: cytomegalovirus
CsA: cyclosporine A
D: donor
EMB: endomyocardial biopsy
HTx: heart transplantation
IHC: immunohistochemistry
PCR: polymerase chain reaction
R: recipient
TRS: total rejection score
